# Anorexia nervosa and hyperphagic episodes : About five clinical cases

**DOI:** 10.1192/j.eurpsy.2024.1156

**Published:** 2024-08-27

**Authors:** I. Belabbes

**Affiliations:** hospital arazi, sale, Morocco

## Abstract

**Introduction:**

Eating disorders affect almost one million people in France. More than half of them have not been screened for the disorder, and are still unable to access treatment!

**Objectives:**

To shed light on the clinical characteristics and management of patients with eating disorders

**Methods:**

We report on a series of clinical situations involving patients presenting with binge eating disorder at the adolescent unit of the Gonesse hospital.

**Results:**

Our sample included 5 patients, all female, aged between 13 and 16 years. They presented with anorexia nervosa with or without hyperphagia. Comorbidities included depression, anxiety disorders, chronic illness and suicidality.

In some cases, treatment is based on re-feeding via a nasogastric tube. In others, behavioral treatment was sufficient. Pharmacological treatment for comorbidities was prescribed.

**Conclusions:**

Untreated eating disorders can be a source of deterioration in patients’ quality of life and high mortality. Early detection and diagnosis is essential for better patient management.

**Disclosure of Interest:**

None Declared

